# Opening the ‘implementation black-box’ of the user fee exemption policy for caesarean section in Benin: a realist evaluation

**DOI:** 10.1093/heapol/czz146

**Published:** 2019-11-20

**Authors:** Jean-Paul Dossou, Vincent De Brouwere, Sara Van Belle, Bruno Marchal

**Affiliations:** 1 Centre de Recherche en Reproduction Humaine et en Démographie, CNHU/HKM, Avenue Jean-Paul II, Cotonou, Benin; 2 Unit of Health Services Organization, Department of Public Health, Institute of Tropical Medicine, 155 Nationalestraat, 2000 Antwerp, Belgium; 3 Health Policy Unit, Department of Public Health, Institute of Tropical Medicine, 155 Nationalestraat, 2000 Antwerp, Belgium; 4 School of Public Health, University of the Western Cape, Robert Sobukwe Road, Bellville 7535, Republic of South Africa; 5 Health Systems Unit, Department of Public Health, Institute of Tropical Medicine, 155 Nationalestraat, 2000 Antwerp, Belgium

**Keywords:** Public/private, hospitals, health policy, user fees, exemption mechanisms, policy implementation, evaluation, theory

## Abstract

To improve access to maternal health services, Benin introduced in 2009 a user fee exemption policy for caesarean sections. Similar to other low- and middle-income countries, its implementation showed mixed results. Our study aimed at understanding why and in which circumstances the implementation of this policy in hospitals succeeded or failed. We adopted the realist evaluation approach and tested the initial programme theory through a multiple embedded case study design. We selected two hospitals with contrastive outcomes. We used data from 52 semi-structured interviews, a patient exit survey, a costing study of caesarean section and an analysis of financial flows. In the analysis, we used the intervention-context-actor-mechanism-outcome configuration heuristic. We identified two main causal pathways. First, in the state-owned hospital, which has a public-oriented but administrative management system, and where citizens demand accountability through various channels, the implementation process was effective. In the non-state-owned hospital, managers were guided by organizational financial interests more than by the inherent social value of the policy, there was a perceived lack of enforcement and the implementation was poor. We found that trust, perceived coercion, adherence to policy goals, perceived financial incentives and fairness in their allocation drive compliance, persuasion, positive responses to incentives and self-efficacy at the operational level to generate the policy implementation outcomes. Compliance with the policy depended on enforcement by hierarchical authority and bottom-up pressure. Persuasion depended on the alignment of the policy with personal and organizational values. Incentives may determine the adoption if they influence the local stakeholder’s revenue are trustworthy and perceived as fairly allocated. Failure to anticipate the differential responses of implementers will prevent the proper implementation of user fee exemption policies and similar universal health coverage reforms.

**Key Messages**

- The implementation of the user fee exemption policy for caesarean section may result in mixed outcomes at hospital level within the same country because of contextual factors such as the ownership of the facility, the organizational objectives of the facility, the commitment of managers, the management of health providers, the existence of channels for community voice and of means for exercising that voice.
- The implementation outcome of such policies may be generated by different mechanisms, including trust, perceived coercion, intrinsic adherence to the goals and values of the policy, perceived individual or organizational financial gains provided by the policy and the perceived fairness in the resources allocation.
- The policymakers should consider strategically which mix of mechanisms they need to trigger in which contexts for a successful implementation of user fee exemption policies or other similar health-financing reforms for universal health coverage.

## Introduction

Since more than a decade, universal health coverage (UHC) is a priority on the global health agenda (Horton and Das, 2015). World Health Organization defines UHC as ensuring that ‘all people and communities can use the promotive, preventive, curative, rehabilitative and palliative health services they need, of sufficient quality to be effective, while also ensuring that the use of these services does not expose the user to financial hardship’ (World Health Organization, 2015). The momentum for UHC coincides with a growing consensus that user fees constitute a barrier to timely access to life-saving interventions for vulnerable populations and may cause financial hardship (Russell and Gilson, 1997; Richard *et al.*, 2008; Arsenault *et al.*, 2013). In theory, user fee exemption policies ‘place an additional resource in people’s wallets. (…) It gives them the opportunity to use public health services whenever they feel the need, without being dissuaded by cost’ (Robert *et al.*, 2017, p. 2). The Lancet Commission on Investing in Health endorsed the progressive universalism approach defined as the ‘determination to include people who are poor from the beginning’ (Gwatkin and Ergo, 2011, p. 2161) in the pathway towards achieving UHC, and stated this requires user fee exemption policies, either without (type one) or with (type two) targeting of poor people (Jamison *et al.*, 2015).

Several African countries introduced user fee exemption policies for various health services since 2000 (Witter, 2010; Richard *et al.*, 2013; Dzakpasu *et al.*, 2014), aiming at promoting equitable access and decreasing catastrophic expenditure (Gilson *et al.*, 2000; Richard *et al.*, 2008, 2013; Ridde, Haddad, *et al.*, 2013; Dossou *et al.*, 2018). Most studies of the implementation of these policies reported that patients continued to be charged fees. Poorly formulated policies combined with insufficient or irregular reimbursements contributed to providers keeping charging fees (Gilson and McIntyre, 2005; Witter and Adjei, 2007; Agyepong and Nagai, 2011). Also the discretionary power of frontline workers has been frequently reported as a major determinant of policy implementation (Walker and Gilson, 2004; Gilson and McIntyre, 2005; Witter and Adjei, 2007; Agyepong and Nagai, 2011; Ben Ameur *et al.*, 2012; McPake *et al.*, 2013; Ridde, Kouanda, *et al.*, 2013). However, little is known on when and why implementation of user fee exemption policies succeeds or fails.

Government of Benin introduced the user fee exemption policy for caesarean section on 22 December 2008, targeting all pregnant women requiring a caesarean section. It came officially into effect on 1 April 2009 and covered an intravenous infusion before referral, referral within the health district, consultation, hospitalization, surgery, drugs, supplies and post-surgery check-up. A national agency (the implementing agency) was created to manage the implementation process. In 2013, 30 state-owned hospitals and 18 non-state-owned hospitals were accredited for reimbursement under the policy. Criteria for the accreditation of hospitals included the not-for-profit status and the capacity to provide caesarean section. The state-owned hospitals included all the hospitals, operating under the administrative and technical supervision of the Ministry of Health (Présidence de la République du Bénin, 2005). The non-state-owned hospitals included facilities administratively independent from the Ministry of Health (Présidence de la République du Bénin, 2005). All accredited facilities received a fixed sum of XOF 100 000 (US$196) for each caesarean section performed, regardless of their level and the actual costs related to the services (Witter *et al.*, 2016). In addition to the reimbursements, the Ministry of Health provided free kits of drugs and consumables during the first year.

The FEMHealth research programme ran from January 2011 to December 2013 and was funded under the Seventh Framework Programme of the European Commission. It aimed at evaluating user fee exemption policies for maternal health services in Mali, Burkina Faso, Morocco and Benin. In Benin, FEMHealth focused on the user fee exemption policies for caesarean section and demonstrated variable implementation outcomes across seven hospitals. Some hospitals provided five out of the eight items covered by the policy for free, whereas other facilities provided only one. The median fees charged for a caesarean section varied between US$0 and US$52 (Centre de Recherche en Reproduction Humaine et en Démographie, 2014; Dossou *et al.*, 2018). In this article, we present the results of a study that aimed at understanding why and in which circumstances the implementation of the fee exemption policy succeeded or failed. With this study, we aim to reduce the literature gap on the implementation of user fee exemption policies in low- and middle-income countries (LMICs). We expect the evidence generated will be relevant for makers, implementers and evaluators of user fee exemption policies and of other UHC policies in LMICs.

## Methods

We adopted the realist evaluation approach, which is a particular form of theory-driven evaluation (Stern *et al.*, 2012), is rooted in realist philosophy of science. At its centre is the notion of generative causation (Pawson and Tilley, 1997; Wong *et al.*, 2016) which makes it particularly adapted to study complex problems (Westhorp, 2013; Bamberger *et al.*, 2016), including in health policy and system research (Gilson, 2012; Adams *et al.*, 2016). The variable implementation of the user fee exemption policy for caesarean section across hospitals is best considered as a complex problem. Indeed, policies are implemented in health systems which are best understood as open systems. Policy implementation is likely to be influenced by existing and new health policies but also by other political, economic and social events. Many actors are involved, from various positions and with specific interests and ideas about the policy (Van Belle *et al.*, 2017). All these point to the complex nature of the causal processes underlying the outcomes of the policy. Realist approach demands the researcher to make explicit the causal mechanisms that explain the observed outcomes and its variation (McLaughlin, 1987; Pawson and Tilley, 1997; Pawson, 2004; Wong *et al.*, 2016). Realist evaluation indeed considers that it is not the policy, but the actors who are engaged in it who bring about the results, situated as they are in specific contexts. Policies work (or not) because actors make particular decisions in response to the resources or opportunities that the policy provides. Realist evaluators thus aim at identifying the underlying ‘reasoning and resources’ (Pawson and Tilley, 1997, p. 68) or generative mechanisms that explain how the outcomes were brought about and in which conditions (Pawson and Tilley, 1997). As such, realist evaluation fits policy implementation research well: it allows for exploring not only the policy formulation and priority-setting process, but also—like in our research question—how and why actors, within the organizational setting and in the broader societal context, implement policies (or not; Marchal *et al.*, 2018). In the following paragraphs, we present the development of the initial programme theory, the study design used to test it, and the methods to collect, to analyse the data and to refine the initial programme theory.

### Development and formulation of the initial programme theory

Realist evaluation starts with eliciting the initial programme theory of the intervention in question (Marchal *et al.*, 2012). The initial programme theory can be considered as the hypothesis that posits how and why a particular intervention would lead to specific outcomes, for whom and in which conditions. Often, the initial programme theory is based on the results of literature reviews. We started with the berry-picking method to explore the field, followed by a non-systematic review of the policy implementation literature. Berry-picking approach is appropriate for identifying elements of the initial programme theory (Finfgeld-Connett and Johnson, 2013). Like realist synthesis (Pawson *et al.*, 2005), the berry-picking method is an iterative, adaptive, creative and flexible process that can contribute to generate a theory that explains a social phenomenon by consolidating or federating fragmented pieces of the literature (Booth, 2008). The first step is a literature search, followed by the selection of a first set of articles relevant to the research question. Analysis of the selected publications leads to the reformulation of the research question, which in turn is followed by a second search and so forth until the synthesis of the results adequately answers the research questions (Booth, 2008). First, we searched for frameworks, models or theories that could inform our initial programme theory. We reviewed papers dealing with the implementation of user fee exemption policies for maternal healthcare and healthcare in general. We found that most publications describe policy implementation gaps and factors that may explain implementation failure, like inadequate inputs and poor communication (Witter and Adjei, 2007; Agyepong and Nagai, 2011). Few studies started from an explicit theory or theoretical framework on policy adoption or implementation. Few authors attempted to explain the success or failure of the policy implementation by using theories or models from political science (public administration and policy analysis). Authors like Ridde and Diarra (2009) and Walker and Gilson (2004) used the concept of street-level bureaucracy (Lipsky, 1980) to explain the implementation gap of user fee exemption policies. However, none of the papers referred to the broader policy implementation literature.

The scant results of the first review led us to review the policy implementation literature. Given the limited consensus on policy implementation theories (O’Toole, 2004; DeGroff and Cargo, 2009), a systematic review was not likely to be useful. We, therefore, carried out a non-systematic review of the published and grey literature. We started the search using the Web of Science and Social Sciences Citation Index search engines with sets of keywords combining ‘policy’, ‘program’ and ‘implementation’ or ‘adoption’. We used extensive snowballing to track down the original papers and books from the bibliographic references of the initial list of papers and books.

This review identified a first set of three publications. We selected the paper of Berman on macro- and micro-implementation of social policy (Berman, 1978) because it presents a comprehensive model, going from policy design to local implementation and back, and it emphasizes the multi-level nature of policy implementation. Second, we selected Elmore’s forward and backward mapping model (Elmore, 1982). This model identifies local agents as key actors in policy implementation and provides an advanced framework for successful ‘planning’ of policy implementation, from the first choices to the expected outcomes (forward mapping) and back (backward mapping). Third, we added the ambiguity-conflict model of policy implementation of Matland, because it represents a theory explaining implementation gaps that goes beyond the top-down and bottom-up divide (Matland, 1995).

At this stage, we extracted from those papers, the contents related to intervention (*in casu* policy), context, actors, mechanisms and outcomes, using the Intervention-Context-Actor-Mechanism-Outcome (ICAMO) heuristic to identify the configurations that explain the reported outcomes at each level (Van Belle, 2014; Marchal *et al.*, 2018). ICAMO is a modified version of the context-mechanism-outcome configuration (Pawson and Tilley, 1997) that stimulates a proper description of the actual intervention and ‘the actors through whom the intervention works’ (Mukumbang *et al.*, 2018). This process pointed us to three ‘passages’ or implementation phases (Berman, 1978, p. 13) that provided the overall multilevel structure of our initial programme theory:

Phase 1: This phase includes the actual decision-making process and the institution of a programme to implement the policy. It involves macro-level key stakeholders, including the ministers’ council, and results in making the decisions for the policy and its financing. Next, an administration or government agency is set up to develop a programme to implement the policy decision. The more ambiguous the policy intent, the more latitude such agency has in shaping the policy.

Phase 2: This phase encompasses the transition from programme to local adoption at operational health service level, during which slippage between programme guidelines and actual implementation can occur. This is partly determined by the degree of compliance with decisions by higher authorities (and thus the enforcement capacity) and alignment of the policy goals with local needs. Programmes are actually or symbolically adopted by district health authorities and hospital managers.

Phase 3: This phase concerns the implementation of the programme by service providers, who act as street-level bureaucrats. Berman calls this ‘micro-implementation’ and considers this ‘may be the most pivotal step because a social policy’s outcome depends on local delivery’ (Berman, 1978, p. 21). Service providers can implement the policy in four ways: (1) non-implementation, (2) co-optation (or adaptation of the programme to fit existing practices), (3) technological learning (adaptation of routine practices to accommodate the policy) and (4) mutual adaptation, whereby both policy and service delivery are adapted to ensure optimal fit. In previous studies, Berman found that only mutual adaptation leads to achievement of the intended policy outcomes: the policy is adapted to the organization, its staff, its target public and its environment, and organizational changes are made to better implement the policy.

**Figure 1:**
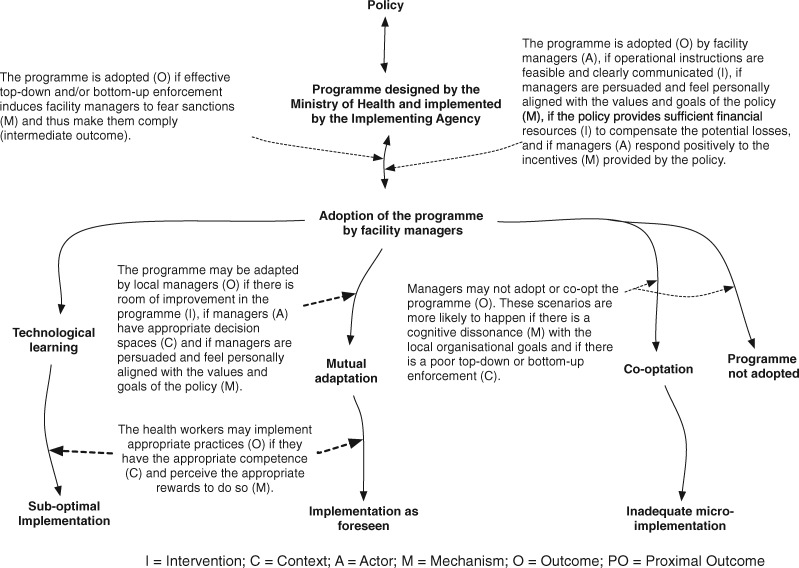
Initial programme theory.

If the three papers provided an overarching architecture for a multi-level analysis of policy implementation, they did not present mechanisms. Our berry-picking approach led to the ‘carrots, sticks and sermons’ typology of policy instruments (Bemelmans-Videc and Vedung, 1998). Carrots, sticks and sermons can be considered as a parsimonious typology of mechanisms underlying policy instruments and more specifically as categories of drivers of individual or organizational commitment to the policy. Policies that include incentives (‘carrots’) may induce a positive response when actors perceive a financial or other gain, or if any financial losses due to the policy are compensated properly (net benefit or no net loss). Policies that are based on coercion and sanctions (‘sticks’) may induce perceived pressure and fear of sanctions that in turn may generate compliance. Policies based on persuasion (‘sermons’) trigger good implementation if the actors perceive an alignment between personal believes and policy goals and values. Figure 1 presents our initial programme theory.

### Study design

We adopted the multiple embedded case study design (Yin, 2009), which is well adapted to research on policy implementation gaps. We defined ‘the policy implementation’ as the case. We considered a hospital implementing the user fee exemption policy for caesarean section as the unit of analysis. Two units of analysis were purposively selected among the seven implementing hospitals covered by the FEMHealth research programme in Benin—a faith-based (non-state-owned) hospital and a public (state-owned) hospital—because they present contrasts in terms of ownership, location and policy implementation outcomes (Table 1). A detailed description of the sampling procedure of FEMHealth is reported elsewhere (FEMHealth, 2014).

**Table 1.** General characteristic of the two units of analysis in 2011

Parameters	The faith-based hospital	The State-owned or public hospital
Owner	A Christian religious organization	State
Population in catchment area (2011)	299 407	247 028
Monetary poverty index in the region of the catchment area (2011) (Biaou *et al.*, 2015)	25.43%	46.07%
Type of hospital	First referral hospital, non-district hospital	First referral hospital, district hospital
Location	Urban area	Semi-rural
Number of beds (2011)	23	80
Key staff of the maternity (2011)	1 gynaecologist,3 midwives	1 gynaecologist,3 midwives
Wards/units	Surgery, medicine, gynaecology, paediatrics, laboratory	Surgery, medicine, gynaecology, paediatrics, laboratory, radiography service
Number of CSs in 2011 over the number of deliveries (percentage)	529/879 (60%)	259/1079 (24%)

*Source:* Administrative records of the hospitals and Biaou *et al.* (2015).

### Data collection methods

Realist evaluation is method-neutral: the data collection methods should allow to gather data needed to test the initial programme theory. We collected both quantitative and qualitative data to describe the case and its units of analysis. Table 2 presents the data sources, tools and their specific purposes. A multi-disciplinary team (a medical doctor, a health economist and a social scientist) collected the data between March 2012 and March 2013.

**Table 2.** Data collection techniques and data sources and study sample in the two study sites

Tools and their specific purposes	Sources and sample from the faith-based hospital	Sources and sample from the public hospital	Analysis of the data
Exit survey To assess the implementation outcome using the remaining user fee paid for items normally covered by the policy.	28 women who underwent a caesarean section.	17 women who underwent a caesarean section.	We entered the data into an excel sheet and identified the median (because the data distribution was not normal), the maximum and the minimum values, presented in a narrative format in the findings.
Financial flow tracking To assess the adequacy of the financial resources provided by the policy to the implementing hospitals. This provides data on a critical aspect of the intervention.	Review of policy documents, financial reports of the implementing agency and of the implementing hospital.	Review of policy documents, financial reports of the implementing agency and of the implementing hospital.	
Caesarean section costing To quantify the direct cost of the resources engaged by the hospital to produce caesarean section. This provides data on a critical aspect of the context.	Interviews with three health providers and two managers to describe the caesarean section production usual practices and validate the caesarean section cost lines; data from medical records of 30 women who underwent a caesarean section have been used to compute the cost.	Interviews with three health providers and two managers to describe the caesarean section production usual practices and validate the caesarean section cost lines; data from medical records of 30 women who underwent a caesarean section have been used to compute the cost.	We entered the data into an excel sheet. The mean production cost has been computed per facility and presented in the findings in a narrative format.
Semi-structured interviews To collect data about (1) the context (existing hierarchical and administrative arrangements, financial challenges of the hospital, human resources management practices, practices in terms of caesarean section service delivery), (2) the intervention (actual implemented practices) and (3) the actors (identification and profile). Data on the reasonings and the interpretation of actors on how the outcome can be explained. Researchers listen first to the explanation of managers, then share their theory and collect comment of actors on them.	Four health district managers, 15 hospital managers and healthcare providers, 6 community representatives in the local health committees, 6 women with caesarean section.	Four health district managers, 8 hospital managers and healthcare providers, 3 community representatives in the local health committees, 6 women with caesarean section.	All the interviews have been transcribed and entered in a QSR NVIVO 10 database. The analysis followed thematic analysis principles. Initial coding was based on a coding framework that drew upon the key elements of the initial programme theory and which evolved during the analysis process. Findings are presented with verbatims.

### Data analysis and refinement of the initial programme theory

We transcribed verbatim the recordings of the interviews and entered the transcripts in QSR NVIVO 10, converted later to QSR NVIVO 12. In a first phase, we adopted thematic analysis principles (Miles and Huberman, 1984), whereby the initial coding was based on the key elements of the initial programme theory, which evolved during the analytical process. We used Microsoft Excel 2011 (version 14.6.7) to do a univariate descriptive analysis of the quantitative data from the exit survey and from the financial flow tracking study. Table 2 presents the analytical approach used for each type of data.

In a second phase, we triangulated qualitative and quantitative data to develop a configurational analysis of each case using the ICAMO heuristic tool (Van Belle, 2014; Marchal *et al.*, 2018). We described the intervention (*in casu* policy), context, actors, mechanisms and outcomes, and identified the configurations that explain the reported outcomes at each level. We mapped the causal configuration looking for causal links between the actually implemented intervention, relevant contextual elements, the mechanisms that were triggered for specific groups of actors and the observed outcomes. Mechanisms are key components in this heuristic and we defined them as the reasoning of the actors in response to the resources or opportunities and changes in the context introduced by the intervention (Pawson and Tilley, 1997). We used a retroductive mode of inference in which the researcher starts from the events observed to postulate the conditions without which those events cannot exist (Meyer and Lunnay, 2013; Robert *et al.*, 2017; Mukumbang *et al.*, 2018). At this stage, the results from the qualitative data analysis were combined with quantitative data from the costing study and the assessment of the policy outcomes.

Once the in-case analysis was finished, we conducted a cross-case analysis to compare the configurations. This allowed us to challenge the initial programme theory, look for alternative explanations and refine the PT, using the ‘If …, then …, because … ’ statements (Van Belle, 2014).

### Ethical considerations

The Benin National Committee for Health Ethics (ref. 0792/MS/DC/SGM/DFRS/SRAO/SA) in 2012, and the respective research ethics committee of the authors’ institutes approved this study. We obtained a written authorization to conduct the study from the Ministry of Health of Benin but also written consent from the managers of each unit of analysis. All the interviewees signed an informed consent form before the interviews. All the materials are stored for confidentiality and the findings are reported anonymously.

## Results

In this section, we present the results of the two hospitals, describing the implementation outcome and the respective responses of the actors: the adoption of the policy by the managers, the response of the providers and the perceptions of the community. We present a synthesis of the ICAMO of both hospitals.

### The faith-based hospital

#### Implementation outcome

In the faith-based hospital, the median fee paid by users for a caesarean section amounted to US$53 (min. US$50; max. US$149). Out of the eight items of the policy package, only the post-surgery check-up was fully exempted.

#### Adoption of the policy by the hospital management team

This hospital was accredited as an implementing facility in December 2008. Six weeks after the official launch of the policy on 1 April 2009, the hospital managers started implementing it. We found that managers of this facility felt they were not properly engaged in the policy design process and that their concerns were not properly addressed. Managers also reported that they did not trust government to timely reimburse the US$196 for each caesarean section as stated in the official policy documents. Managers justified this mistrust by previous experience with government:

*We used to exempt some user fees for civil servants affiliated to the national health insurance scheme, but the state never reimbursed us. So, when we received the information [to implement the fee exemption policy], we were a little bit hesitant. We waited for other facilities to start. We started after seeing that the reimbursement was effectively following* (Hospital manager, 2012).

Our financial flow tracking study showed that up to December 2011, this facility was reimbursed US$205 800, corresponding to 1050 caesarean sections. Our respondents declared that it took between a few days and 3 months after submitting the claims to receive the reimbursements.

Several reasons were given for not fully adopting the policy. The managers perceived the fixed amount of US$196 insufficient and unfair. Yet, the analysis of the costing data showed that the median production cost of a caesarean section (including all the direct costs) was US$50 (min. US$41; max. US$76). User fee charged in this hospital before the policy was US$180. Managers argued that their reimbursement rate should be higher than that for the state-owned facilities where public subsidies cover some recurrent expenditure. They, therefore, decided to systematically charge patients an additional fee of US$39. Non-exemption of consultation fees was explained as follows:

*Even when a pregnant woman later undergoes a caesarean section in the facility, she has to pay the consultation fee before being admitted to the hospital. (…) since you cannot predict if the woman will undergo a caesarean section or not, and thus if she has to be exempted for the consultation fee or not. Gynaecologists are very strict on this point, because their income depends on this* (Hospital manager, 2012).

In this hospital, gynaecologists receive 5–10% of the fee of each caesarean section they perform. To receive this fee, they have to present a proof of payment of the additional US$39 fee to the administration. Gynaecologist declared they have to ask actively women to pay the fee and collect the proofs they can show to the administration. Users declared they feel constant pressure from health workers to pay the additional fee.

In addition to the reimbursements, the Ministry of Health provided free kits of drugs and consumables during the first year of the policy. Kits were issued to the maternity, where they were used for patients who paid the additional fee. The managers interpreted the provision of kits as a permanent measure. When it ended in March 2010, they considered this an important loss of resources and they started charging users all the post-operative care, on top of the additional US$39 additional fee. They reported this change to the implementing agency during a supervision visit. The agency expressed its dissatisfaction and issued an injunction to start implementing the policy as requested.

*There are pre-anaesthesia consultations that we charge the patients for and that was noticed by the implementing agency when they visited the hospital. They were angry, they really shouted, even in front of patients* (Health worker, faith-based hospital, 2012).

The managers, however, did not adhere to the injunction. We found that the managers as well as the providers perceived the hierarchical power of the Ministry of Health as weaker than that of the religious organization owning this hospital. We found that the latter controls the management of the hospital through the appointment of the managers, the recruitment of the staff and the validation of the budget.

#### The response of the providers

The providers reported that they adopted the package of the policy only partially. It emerged from the interviews that communication between managers and providers was limited. Providers expressed their feeling that their managers had a laisser-faire attitude about the policy. Providers said they received little organizational or supervision support as illustrated by the following quote:

*(When the implementing agency staff were critical), the managers did not support us. The gynaecologist who was supposed to be responsible of everything related to caesarean sections was blamed for all that went wrong* (Health worker, faith-based hospital, 2012).

The supervision visits from the implementing agency were irregular and health providers perceived these visits merely as a control of prescription of drugs and consumables. They felt the visits did not help them in finding solutions to the problems they faced in implementation.

Some policy beneficiaries reported in interviews that providers charged them for certain services related to caesarean sections without bill. Providers also reported that they had margins of freedom in the policy implementation that allowed them for instance to prescribe drugs or consumables that were out-of-stock or missing in the kit. As a result, patients had to purchase these items either outside of the facility in private pharmacies or inside the facility (informally from certain providers) as reported in the following quote:

*We sell our products. We prescribe certain drugs. When users buy them, (…) we use them. When they don’t find them, we help them. Usually this applies to hyperbaric bupivacaine or spinal needles. In the last three months, there was no ephedrine at the Essential Drugs Purchasing Centre, nor on the market, but we have our own supply chains* (Health worker, faith-based hospital, 2012).

We found that there was little if any contestation of these practices by patients.

#### The view of the community

The community representatives in the district health committee (*Comité de Santé*) reported in interviews that they had little knowledge about the policy and were not much engaged in it. The committee is supposed to ensure participation of all stakeholders in local health governance as prescribed by decree N° 2005-611 of 28-09-2005 (Présidence de la République du Bénin, 2005). Yet, in 2012, 3 years after the start of the policy, some key members of the committee reported they were still not aware that the hospital was receiving government funding to implement the policy. Consequently, the community representatives mentioned they did not exert pressure on the managers nor on providers to exempt all fees as requested by the policy.

Our interviews also showed that some patients did not know that the policy applied to this hospital when they were admitted. They reported also not knowing the exact package of exempted services and how the hospital implemented this.

Managers, providers, users and community representatives reported there were no formal channels, such as complaints procedures, for patients to engage with the management team. Patients who were still charged fees did not actively engage with local health managers, hospital managers nor providers to demand the policy to be implemented. When asked why, patients mentioned they perceived this facility as a faith-based hospital that provides quality care inspired by religious, social and humanitarian values, and that they trusted the management team.

#### Synthesis of the findings in the faith-based hospital

**Figure 2:**
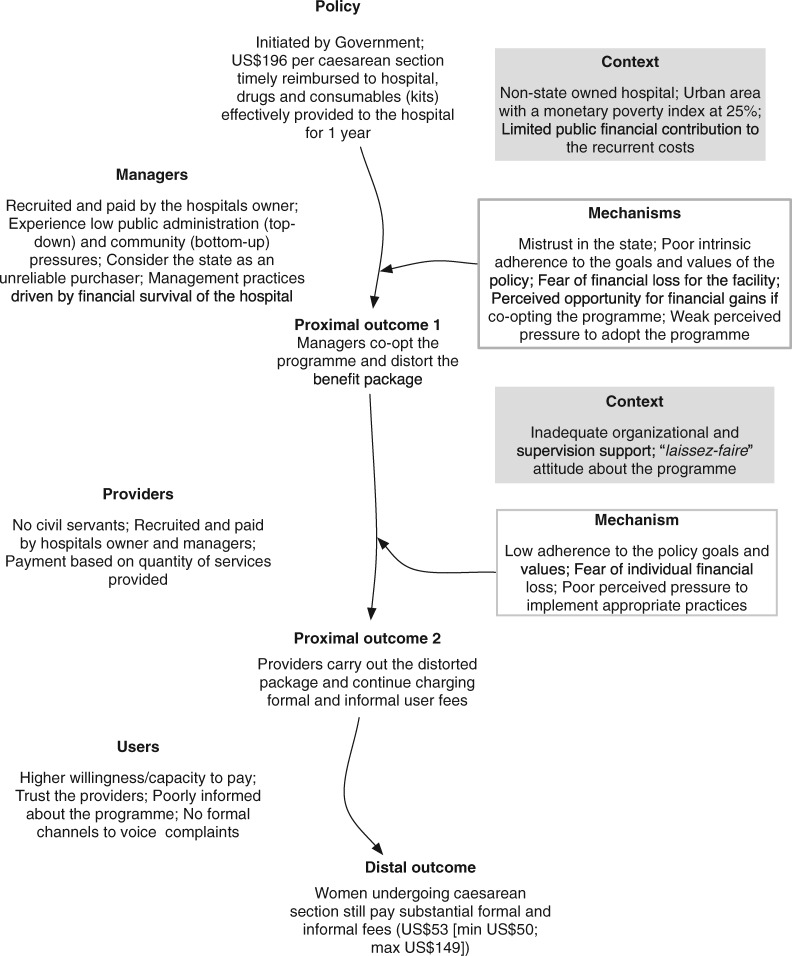
ICAMO configuration in the faith-based hospital.

At this hospital, patients still paid for a caesarean section after the policy was formally adopted by the management team. In actual practice, the managers did not implement the policy as requested: mistrust in the state, concerns about financial survival of the facility, perceived unfairness in the relative allocation of resources to state-owned and non-state-owned hospitals, and the weak perceived top-down and bottom-up pressure to implement the policy contributed to their decision. Poor communication, poor organizational and supervision support, and a laisser-faire attitude contributed to health professionals exploiting opportunities to charge informal fees that impacted negatively on policy outcome. Figure 2 presents the ICAMO configuration that summarizes the findings.

### The state-owned hospital

#### Implementation outcome

In this hospital, patients were fully exempted for five of the eight items of the caesarean section package. The median user fee for this package amounted to US$7 (min. US$0.2; max. US$59).

#### Adoption of the policy by the management team

Managers of this hospital applied the policy on 3 April 2009, 2 days after its official launch. The hospital director was involved in the technical workshops organiszed during the development phase of the policy. He perceived the policy as supporting his vision to increase financial accessibility of the hospital. He took active measures to facilitate its implementation, as explained by a manager of this facility:

*We have sensitized healthcare providers and the community. (…) We started exploring what to put in place to prevent the failure of the policy, for instance, by ensuring a sufficient stock of rehydration fluids for a minimum of six months (…) Our vision was that if the government is out of breath, we should be able to continue* (Manager, the state-owned hospital, 2012).

The managers used radio programmes as well as informal communication channels to inform the community about the policy. They simplified the message to facilitate the communication: ‘Caesarean sections are fully free’.

In this facility, managers are civil servants who are recruited, appointed and paid by government. They expressed how the power of the hierarchy within the Ministry of Health made them implement the policy. The perceived power of the community added to this pressure:

*The population has been widely informed that the caesarean section is free and it is difficult to say now “Me, I will not start implementing the policy”. (…) Since the Provincial Health Director knows that the caesarean section is free, if a woman complains that the facility asked her to pay, as a manager, you will have to explain why* (Manager, the state-owned hospital, 2012).

The managers considered the guidelines of the implementing agency regarding reimbursement as useful. Managers felt effectively supported by the implementing agency and this helped in accelerating the reimbursement. Our financial flow tracking study showed that up to December 2011, this facility was reimbursed US$134 652 for the 687 caesarean sections it performed.

#### Response of the providers

In the interviews, providers declared that the hospital management team effectively supported them in the implementation of the policy, for instance with the regular supply of the maternity unit with the caesarean section kits. Managers reported that when they anticipate a shortage of key products of the kits, they give priority to the free caesarean section policy and stop supplying other wards. Women reported that usually they did not have to buy drug in private pharmacies, except when presenting with complications. For instance, in case of meconium-stained amniotic fluid, providers reported that the staff ask families to buy an infusion of metronidazole and ceftriaxone. We found that some non-official fees were charged in the operating theatre and in the post-operative room. For instance, providers proposed selling pain-relieving drugs to women suffering from post-operative pain.

*When the effect of anaesthesia waned, we started feeling uncomfortable and women started crying from everywhere. We were obliged to call the midwives and beg them and ask if they would not have a drug that can reduce our pain. They replied [the managers] said caesarean section is free, and if we want a pain relief, we must pay for it. Then we begged them to put something in our infusion* (Patient, state-owned hospital, 2012).

Both managers and providers reported that the management team did not tolerate such practices. Managers reported that they applied two measures when they found out: the confiscation of drug stocks belonging to staff and the reimbursement of the users who complained. The implementing agency’s team, which conducts supervision visits, sometimes participated in seizing drugs from staff.

#### The view of the community

The district health committee includes community representatives who play a leadership role in the management of the hospital, which is considered by the local communities as ‘our hospital’. The committee meets at least three times per year. In interviews, members of this committee reported the policy was a major event for them: they were informed about the policy and closely communicated with the community to inform them.

Managers, providers and users reported the perceived financial gain from the policy was very important, and the population had high expectations of the policy. Managers explained that living in a semi-rural area, the population had few choices in terms of care providers. As their capacity to pay was low, the high financial burden of a caesarean section and the consequences were important, and the perceived gain from the exemption was very important for them.

Not only users and community representatives but also managers mentioned there were multiple opportunities to express complaints. In constituency meetings with local politicians, they could express their (dis)satisfaction by giving a mark to the public health facilities. Local radio stations provided space for complaints through anonymous phone calls. However, women found it difficult to lodge complaints against staff as long as they were hospitalized, as they feared retaliation.

#### Synthesis of the findings in the state-owned hospital

**Figure 3:**
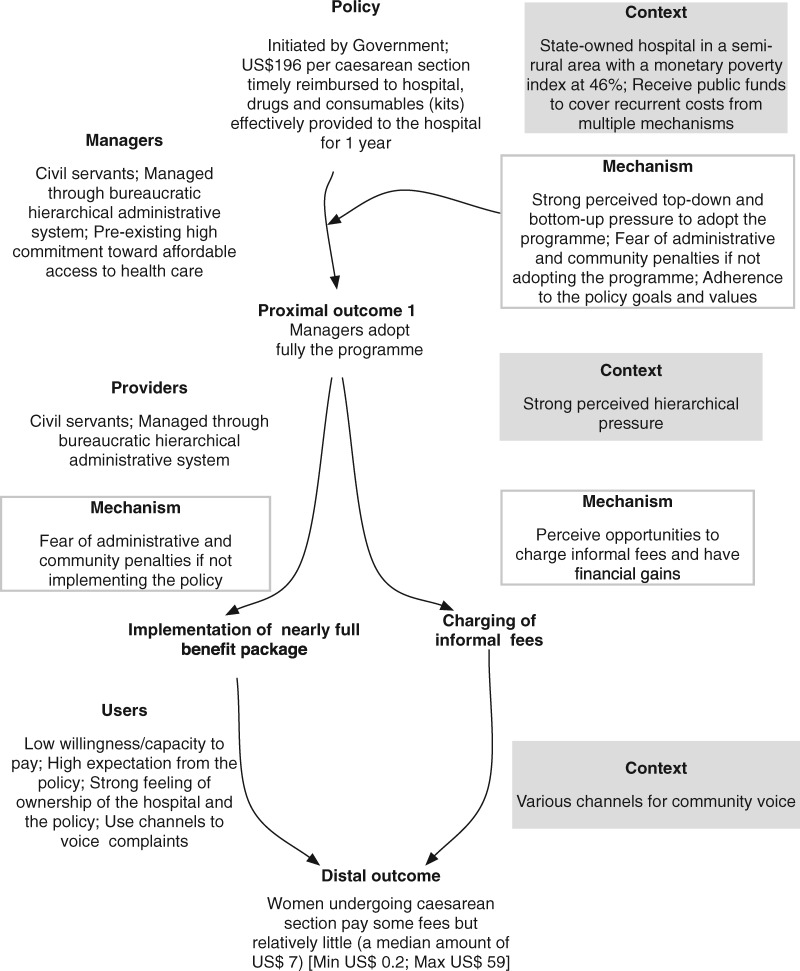
ICAMO configuration in the state-owned hospital.

In this public hospital, patients undergoing a caesarean section still paid formal and informal fees but far less than in the non-state-owned hospital. Managers adopted five out of the eight items of the policy package. They were motivated by their involvement in the development of the policy and by the alignment with their values and principles, but as civil servants, they also felt forced to implement the policy. They actively informed the public and made efforts to support their providers in the implementation. As a result, they adhered to the policy goals. The providers, as civil servants, complied with the policy, and felt actively supported by their managers. The community and users had high expectations of the policy and had channels to raise complaints, exerting bottom-up pressure on providers and managers. Figure 3 presents the ICAMO configuration.

## Discussion

With this study, we aimed to explain the diverging outcomes in two hospitals where the policy was implemented. We focused on the meso- and micro-level of implementation. Several factors were at play in the adoption of the policy, including alignment with personal and organizational goals and values, financial motivation and enforcement. We found new elements not included in our initial programme theory: trust and perceived fairness of the resource allocation. A somehow surprising finding was that the faith-based hospital did not correctly implement the policy, to the detriment of the patients and against the generally assumed value set of such organizations (Olivier *et al.*, 2015). This section is organized around the plausible mechanisms identified, how they operate to generate the outcomes with regards to the resources provided by the policy, the actors and the context? We discuss the strengths and limits of the study and end with the refined programme theory.

### Trust

In the faith-based hospital, there was mistrust of government before the introduction of the policy, which resulted from previous negative experiences with government. This initial mistrust diminished when hospital managers were reassured by the adequacy and timely provision of the kits and the dependability of the reimbursements. This made them feel capable of partially implementing the policy without incurring financial losses. This explanation is consistent with the theory of planned behaviour, according to which individuals are unlikely to develop a strong intention to act and behave in a certain way, if they think they do not have the resources and opportunities to do so, even if they have a positive attitude towards the behaviour in question and if that behaviour would be supported by subjective norms (Ajzen and Fishbein, 1980). For policy implementation, this means administrative injunctions and new norms are unlikely to be adopted if implementers feel they are not enabled to carry it out. Erasmus *et al.* (2017) reported organizational trust is a soft component of systems that policymakers usually overlook but that influences policy implementation.

### Perceived coercion

We found that the policy was better implemented where the perceived pressure was high, either generated through top-down bureaucratic hierarchy or through bottom-up community voice. This pressure depended on the ownership: it was high in the state-owned hospital and low in the faith-based organization, where the authority of the implementing agency was not sufficient to effectively coerce the hospital management team to correctly implement the policy. The implementing agency did not develop a holistic and strategic approach to the accredited facilities (Dossou *et al.*, 2018). In the faith-based hospital, the authority of the Ministry of Health, and thus of the implementing agency, was further weakened by the perception of the state as an unreliable partner.

This calls for further attention to the enforcement capacities of the state. In most LMICs, governments work with mixed health service delivery platforms to deliver UHC (Wadge *et al.*, 2017). In Benin, for instance, five faith-based or association-run hospitals are appointed as district hospitals (Health System 20/20, 2012). However, the governance of such public–private engagements challenges the traditional bureaucratic public administration approaches (Health System 20/20, 2012; SHOPS Project and Inc., 2013; Bertelsmann Stiftung, 2018). In Benin, the Ministry of Health is responsible for governing non-state-owned hospitals, but in practice, its interventions are mainly focused on the initial accreditation. Monitoring, supervision and training are limited and inconsistent. In Benin and elsewhere in Africa, a key issue is how to balance an effective regulatory framework with the managerial autonomy—considered as a key strength—of the non-state-owned facilities (Boulenger and Criel, 2012). State-owned and non-state-owned facilities differ in terms of organizational culture, organizational values, structures and funding. Influence of such differences on the implementation of public health policies remains to be properly addressed in research and policymaking.

The perceived pressure can result also from an active community. Effective governance of local health systems calls for empowered communities capable to raise their voice, claim their rights and hold all providers accountable (Rifkin *et al.*, 1988; Rifkin, 1996; Kenny *et al.*, 2015). In practice, strategies to improve public accountability are often lacking (Van Belle and Mayhew, 2016). Beyond effectively informing the key stakeholders, including the public, and providing formal access to complaint channels, public accountability is a complex process with specific challenges: fragmentation of actions and actors, conflicts of interests, diverging values, and different management and accountability practices (Van Belle and Mayhew, 2016). These structural dimensions have to be addressed properly to prevent, for instance, the persistent informal payments in Benin.

### Intrinsic adherence to the goals and values of the policy

We found that the management team can play a critical role by ensuring a good fit between policy and organizational context. Its commitment to the policy can be triggered by persuasion. Persuasion as a governance instrument is extensively documented in political sciences (O’Keefe, 2015), with the core idea that the lines of authority are getting increasingly blurred and that ‘in such an environment, persuasion skills exert far greater influence over others’ behaviour than formal power structures do’ (Cialdini, 2001, p. 72). In our study sites, government’s capacity of persuasion was different. In the public hospital, the policy’s aim of abolishing user fees clashed with the pro-user fee attitude dominating the Ministry of Health (Dossou *et al.*, 2018). Because the faith-based hospital relied heavily on revenue raised by user fees, the policy contradicted the core organizational goal of survival of the hospital. This leads to cognitive dissonance (Cooper, 2007) at various levels. It explains at least in part why managers kept charging formal fees to users, whereas providers continued charging informal fees.

### Response to the financial incentives and perceived fairness of the resource allocation

The response to the financial incentive within the policy influenced its adoption and the practices of providers. We found that in the faith-based hospital, the perceived financial opportunities made managers to adopt the policy once they saw that the reimbursements were effective and timely. Indeed, the amount reimbursed is 16 US$ higher than the previous tariff charged in this hospital. The accumulated payment of the caesarean section fees at the end of a given period provides a saving fund that increases the investment capacities and constitutes a buffer against loss of revenue due to unpaid bills. However, perceived fairness of the allocation of resources influenced their adoption, too. Indeed, a perceived unfairness of allocation in the sense of a perceived lower subsidy to faith-based hospitals counteracted to some extent the perceived benefit. Perceived fairness was not part of our initial programme theory.

Using incentives to change health systems in LMICs has gained prominence in the last 20 years with various models of result-based financing programmes (Meessen *et al.*, 2011; Soucat *et al.*, 2017). The World Health Organization considers those programmes as a step towards strategic purchasing that encompasses a wide range of governance instruments beyond incentives (World Health Organization, 2018). Our study provides empirical evidence that incentives operate differently in function of the context. Response to incentives may be informed not only by what providers believe to gain individually but also by the comparison with the perceived benefits of other similar providers.

### Strengths and limitations of the study

The realist approach does not link validity to the use of particular methods or tools, but to the coherence between the research objective, the methods used to achieve it, the context and the conclusions (Maxwell, 2012). Maxwell (2012) suggested three dimensions (descriptive, interpretive and theoretical validity) to assess the trustworthiness of the plausible explanations of a social phenomenon offered by a particular study. Regarding descriptive validity, for instance, we relied for the description of the context, the practices and the events on data from different sources, including national policy managers, local managers, providers, users and community representatives in district health committees, policy documents and routine data. One of the limitations of this study is that we did not collect data from other groups such as non-users of the selected hospitals, health providers from other wards or the leaders of the religious organization that owns the faith-based hospital. We attempted to offset this limitation by collecting a wide range of data in order to triangulate findings. A second limitation is that the data we used have been mainly collected in 2012 and 2013 and that the policy context has changed in the meantime. The larger doctoral study of which this article presents some results deals in detail with the changing policy context. To answer the research questions of this study, we believe the temporality plays a lesser role.

### The refined programme theory

**Figure 4:**
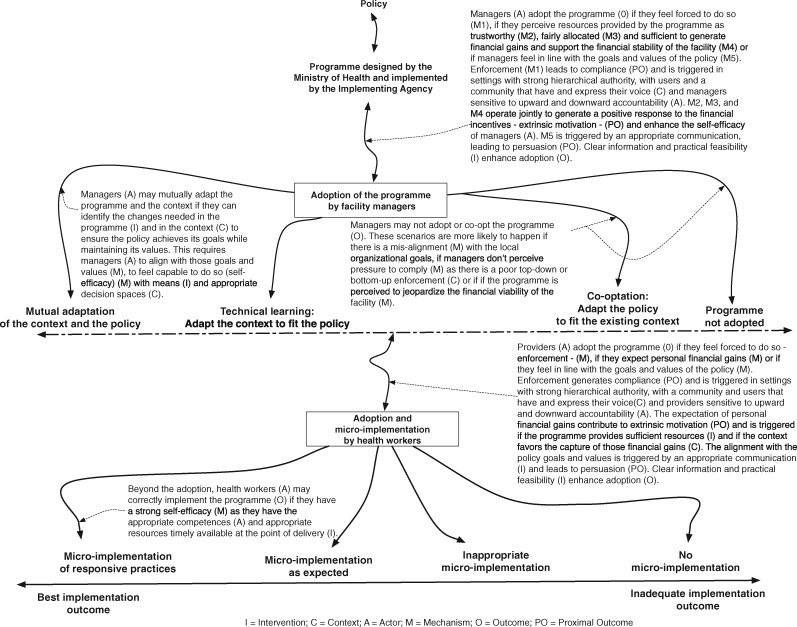
Refined programme theory.

The section below presents the refined programme theory (see also Figure 4).

At the meso and micro-level of the policy implementation chain, the managers of the hospital, and the providers have to adopt the policy. Actual implementation requires the providers to align their practices with the goals of the policy. To be adopted and micro-implemented properly, the policy will have to trigger mechanisms such as trust, perceived pressure, positive response to financial incentives, perceived fairness in the resources allocation and an intrinsic adherence to the policy goals and values in the right mix. Furthermore, the working environment has to be conducive for implementation.

For both managers and providers to adopt the policy and effectively enable its implementation in their hospital, a number of intermediary outcomes need to be achieved.

Compliance is achieved by the perceived pressure that derives from the enforcement of ‘authority’. Hierarchy is an important factor for enforcement and can be enacted through direct command, supervision, control, auditing or the overall accountability procedures. Compliance can also be enforced by the community and users through two components: having a voice (being informed and having effective channels to express their voice) and using that voice (actively expressing voice). The sensitivity of managers and providers to such enforcement depends on their perception on how accountable they have to be towards their hierarchy (upwards accountability) and citizens (downwards accountability) and thus on the degree of pressure exerted on them. Enforcement can be counteracted by perceptions of threats to vested interests, such as institutional survival or informal generation of revenue.

Extrinsic motivation can drive both adoption and micro-implementation. It may be generated by financial incentives. Policies that increase personal or institutional financial gains are more likely to be adopted, but perceived fairness in the allocation of the resources between the parties beyond the net personal financial gains can play a role, too. The perceived financial gains will more likely operate in organizations with lower financial margins and depend on the trust the implementer has in the policy promotor.

Persuasion is effective when the implementers adhere to the policy goals because they became policy champions. The latter will more likely adapt both the policy and the context for an optimal policy implementation. Participation of implementers in the policymaking process can contribute to persuade them, enhance coherence of the policy with their actual needs and increase ownership of the policy.

Effective implementation needs the required resources, including drugs, supplies and information, to be timely available at the point of delivery and a general conducive work setting. These measures, if they are implemented consistently, reinforce trust and enhance the self-efficacy of implementers.

Figure 4 presents a graphical representation of the refined programme theory.

## Conclusions

In Benin, the user fee exemption policy had different implementation outcomes. In the hospitals we studied, contextual factors, including ownership of the facility, organizational objectives, commitment of managers, the management of health providers and the existence of channels for community voice and for exercising that voice transformed the same inputs into contrasting outcomes. In LMICs, trust, perceived coercion, adherence to the policy goals, perceived financial incentives and fairness in their allocation need to be tactically used for a successful implementation of user fee exemption policies—or similar health-financing reforms for UHC—in mixed (state-owned and non-state-owned) delivery platforms.
